# The Viability of Online Pharmacies in COVID-19 Era in Korea

**DOI:** 10.34172/ijhpm.2020.260

**Published:** 2021-01-02

**Authors:** Hyun Sue Song, Byung-Mu Lee

**Affiliations:** ^1^Division of Medical Products Safety, Ministry of Food and Drug Safety, Cheongju, South Korea.; ^2^Division of Toxicology, College of Pharmacy, Sungkyunkwan University, Suwon, South Korea.

## Introduction

 South Korea, officially the Republic of Korea (henceforth referred to as “Korea”), ranks high in e-commerce (4th, 2018), and has competitive digital industry in information and communication technologies (ICT) (2nd, 2017).^1,2^ However, Korea is one of only two countries that have not allowed online pharmacy among the Organisation for Economic Cooperation and Development (OECD) countries. Although ‘doctor-to-doctor’ telemedicine was recognized by the revision of the Medical Service Act in 2003, telemedicine is still not widely implemented in Korea as a result of prohibition of ‘doctor-to-patient’ telemedicine. Likewise, online pharmacies including mail-order sales and pharmacist’s online consultations are strictly prohibited because the sales of medicines to patients can be carried out by pharmacists only at pharmacies under the Pharmaceutical Affairs Act. Telemedicine and online pharmacy are still not launched although the discussion over e-health reformation and online pharmacy has been ongoing since 20 years ago.

 According to the World Trade Organisation report, coronavirus disease 2019 (COVID-19) triggers further digitalization of society and the development of policies for e-commerce, which can be an important solution and an economic driver.^3^ Therefore, many countries are actively expanding telemedicine and online pharmacy (a form of healthcare e-commerce) as part of COVID-19 response. Since telemedicine is an effective countermeasure to prevent the spread of the virus, it can provide remote access to routine care with minimum exposure in a hospital or pharmacy. The United States promoted telemedicine through temporary regulatory relaxation, and made efforts in telepharmacy strategy by Centers for Disease Control and Prevention’s pharmacy guidance.^4,5^ China launched remote pharmacy services such as online drug prescribing, consultation and delivery.^6^ Japan temporarily allowed telemedicine and delivery services for first-time patients.^7^ Although the government-led e-health agenda in 2019 slowed down due to social opposition, Korea also temporarily allowed telemedicine during COVID-19. COVID-19 gives the Korean government paradoxical opportunities to move forward with e-health project.

 This paper is written at the beginning of the paradigm shift to e-health innovation beyond simple institutional changes to prepare for a post-COVID-19 world. In this paper, we discuss the challenges and viability of online pharmacy in Korea. It may also be useful for countries that have not yet allowed online medicine sales.

## Why Has the Korean Government Been Delaying the Launch of Online Pharmacy for 20 Years?

 Korea has higher pharmacy density than average OECD countries and strictly regulates community pharmacies. For example, only pharmacist can limitedly operate one pharmacy, and pharmacy chain is prohibited. Also, there is pharmacy monopoly over prescription and over-the-counter medicine. Pharmacists are obliged to provide consultation of medicine to patients, and any retail trade of medicine outside pharmacy store is forbidden. Similarly, hospitals and doctors are also strictly regulated. For these reasons, there are no big corporations that dominate pharmacies or hospitals in Korea.

 The Korean government enforced ‘the separation of prescribing and dispensing’ and ‘merger into a single national health insurer’ to reform overall health system in 2000. Korean healthcare system is a single-payer National Health Insurance system that covers most of the population and fee-for-service payment system. Moreover, healthcare providers are private-dominated, market-oriented and government-controlled. Due to this system, tension between government and private healthcare providers has been substantial.^8^

 Since the major reform, there have been many attempts to establish telemedicine by government. Having considered the risks and benefits, Korea has also explored the introduction of online pharmacy, and reviewed to amend Article 50 of the Pharmaceutical Affairs Act, which is the legal basis to ban online medicine sales. These attempts, however, were thwarted several times in the face of strong opposition from civil society.

 The survey result about pharmacists and public participation also coincided with the opposition stance of civil society. In a questionnaire survey for pharmacists (with 250 respondents, response rate 70%) conducted in 2012, 53.1% of them opposed to online pharmacy.^9^ After 4 years, in an online survey of public views, 91% of the participants (with 11 796 participants) opposed to online medicine sales with the reasons of substandard and falsified drugs or illegal websites.^10^

 The main reason for civil society’s voice of opposition to online pharmacy seems to be the negative outlook on the outcome of the policy. Koreans are satisfied with the current healthcare system.^11^ In addition, the public feels burdened by the increase in healthcare costs. In fact, Korea has the highest annual average growth in health expenditure per capita among OECD countries over the five-year period.^12^ Koreans are afraid that transformation into e-health system may cause monopoly by big corporations with retail-based pharmacies and clinics, and consequently lead to high healthcare cost. Therefore, high costs might result in the purchase of cheap illegal online drugs. The final outcome may be a threat in the overall public health.

 Despite the Ministry of Food and Drug Safety’s aggressive effort to shut down illegal websites by launching ‘Cybercrime Investigation Task-Force,’ the National Assembly has raised questions about its effectiveness several times. The Korean health authority has been taking a negative position in allowing online sale of medicines to thoroughly monitor borderless illegal drugs unlike trade promotion authority or ICT authority.

 Without the empathy from society, there has been naturally no progress in the past 20 years. That is why Korea has failed for last 20 years despite several attempts to allow online pharmacies to develop the e-health industry.

## Is It A Viable Challenge to Allow Online Sale of Medicines in Korea?

 According to the 2011 World Health Organization (WHO) survey and the 2017 International Pharmaceutical Federation survey, the number of countries allowing online pharmacies has increased.^13,14^ During the pandemic, more people use online pharmacies and more governments encourage people to buy medicines online. Concurrently the Food and Drug Administration and the European Medicines Agency are constantly alerting consumers and actively responding to cyber crime in medical products focused on COVID-19.^15^

 Despite the efforts by Lee’s warning of ‘losing battle against sales of illegal medicines online’ and Mackey’s concerning about ‘digital danger by illicit online pharmacies,’ it may still seem impossible to completely eradicate illegal online drugs.^16,17^

 In spite of the pessimistic perspective, is it a viable challenge for Korea and other countries that have not allowed online sale of medicines to allow it? To evaluate the viability of online pharmacies in Korea, SWOT analysis was conducted as one of the analysis methods widely used for strategic planning.

*Strengths: *The world’s top online environments and infrastructures – high-speed internet, high broadband and smartphone penetration rates, high number of Internet users per population, consumer familiarity with ICT and e-commerce, high e-government index, enough ICT professionals - are Korea’s strengths. Korea has built a digitalized national-level public health record system which can provide healthcare big data open systems. Electronic system on drug track-and-trace is fully implemented throughout the drug supply chain in 2018.

*Weaknesses: *In contexts where the government lacks statistical evidence on effects of online pharmacies affecting pharmacists and patients in Korea, it hardly seems to convince the civil society. There is also insufficient regulation on online pharmacies. For example, government and pharmacist organization could not reach to an agreement to introduce standards on quality pharmacy services, namely the “good pharmacy practice,” necessary to regulate brick-and-mortar and online pharmacies. Given the nature of online infrastructure, online pharmacy policy is efficient when implemented by a single government body, but executive authorities are distributed among four branches of government.

*Opportunities*: There is a high-value market opportunity in Korea’s online pharmacy due to rapid population aging, growing health-consciousness and sharp increase in health spending per capita.^12,18^ In particular, the percentage of pharmaceutical spending in Korea accounts for 20% of health spending, and it is much higher than the OECD average.^19^ Therefore, online pharmacy can be a meaningful solution for ‘medically vulnerable and underserved populations (eg, elderly patients, mobility impairments, rural and remote populations)’ in Korea, which is one of the world’s fastest-aging countries.

 Some favorable factors were given for Korea’s online pharmacy policy in 2020. In response to COVID-19, the Korean government allowed a temporary exception to use telemedicine. As a result, about 327 000 cases of both online doctor visits with prescription and pharmacist consultations by phone were successfully conducted over three months.^20^

 In addition, the government paid a keen attention on healthcare and digital service sector, and announced an investment of about 4 billion dollars until 2023, expecting to open a window of opportunities for not only pharmaceutical and healthcare industries but also new industries.^21^ Furthermore, Korea has adopted changes to the so-called three major data privacy laws, which had the benefit of protection to personal data and the barrier to the development of e-health.^22^ The amendments, effective August 5, 2020, made it possible to utilize of medical data.

*Threats:* The real threats facing Korea, come from risks to unauthorized medicines across borders due to the failure of coordination among ministries. For example, there were non-rapid seizure of illegal website domains, and conflict among laws such as re-sale of import drugs for personal use. The Korean government has so far attempted to effectively oversee medicines distributed across borders over the internet. However, despite the strong internet infrastructure and pharmaceutical regulatory systems in Korea, it is virtually impossible to produce tangible outcomes.

 Meanwhile, considering that the number of doctor’s consultations per capita (1st, 2018) and annual growth in pharmaceutical expenditure (2008-2018, hospital pharmaceuticals 1st; retail pharmaceuticals 2nd) in Korea are very high among OECD countries, increasing usage of telemedicine can deteriorate financial health of National Health Insurance by prescription drugs spending.^12,23^ Besides, increasing visits of online pharmacies can threaten the public health by misuse or overuse of over-the-counter drugs in relation to lacking communication with health professionals and the problem of self-diagnosis.

 In addition, competition between domestic healthcare service companies and experienced global companies may represent drastic changes in Korea’s well-designed healthcare system. It may cause a collapse of community pharmacies (Figure).

**Figure:**
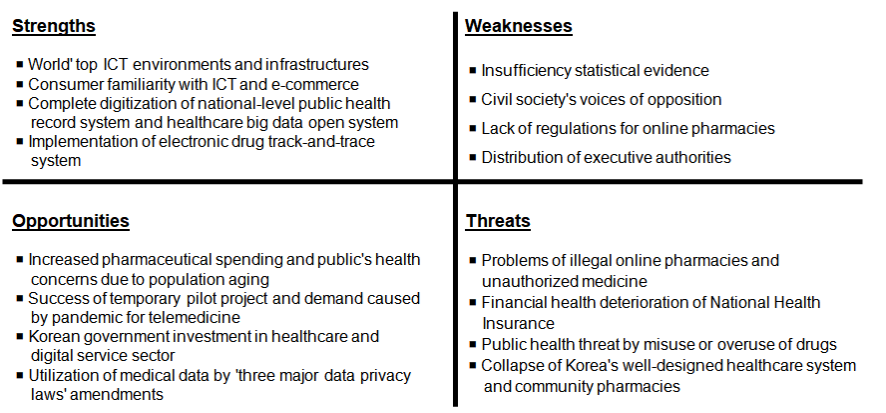
A SWOT Analysis of Online Pharmacy in Korea. Abbreviation: ICT, Information and Communications Technology.

 After COVID-19 outbreak, consumption of overall healthcare sectors has decreased in Korea, causing a damage in the industry of pharmacies and clinics.^24^ Online pharmacies should be the part of routine health system for effective emergency response to pandemics.^25^ At the same time, the revitalization of the industry is needed because online pharmacy market has grown through the expansion of internet.^26^

 In some cases, the government had to recognize the right to sell medicines online in accordance with the court’s decision in dispute (eg, Japan, Germany).^27,28^ This trend and global environment may teach lessons to the Korean government and other countries that have not yet allowed online medicine sales. In other words, the recognition of e-commerce of legitimate pharmacies could be a practical solution from macro perspective in the post-COVID-19 era.

## Conclusion

 Korea has a developed favorable environment for e-health, and the COVID-19 has accelerated the delayed challenge. Currently, Korea’s most important challenge is to gain the support from the public and to prevent possible consumer’s risks caused by allowing online pharmacies. The Korean government will gain consent from the civil society by offering strategies for online pharmacies based on statistics and analysis. These must be obtained through the implementation of temporary telemedicine in response to COVID-19, and the utilization of medical information made possible by the effectuation of ‘three major data privacy laws’ amendments.

 In addition, strategy for online pharmacies, designed by the Korean government, should be presented in various areas such as drug advertisement, provision of drug information, consultation, drug supply chain, relief system for drug adverse reactions, customs clearance, e-commerce, prevention of cybercrime, security and privacy. One of the expected strategies for the Korean government is to acknowledge the online medicine sales only to pharmacy store owners and introduce online pharmacy licensing system, which can be jointly certified and managed by the government and the Korean Pharmaceutical Association.

 Online pharmacy will pose a grand challenge in Korea, but it is also an opportunity for the public to increase the convenience and accessibility for the choice of drugs. It may also enhance the competitiveness of pharmaceutical and healthcare industry. Now that many countries are moving toward active e-health systems, it is an important time to innovate healthcare system in Korea.

## Ethical issues

 Not applicable.

## Competing interests

 Authors declare that they have no competing interests.

## Authors’ contributions

 Both authors were involved in the conception, writing and revision of the paper.

## Disclaimer

 The views expressed in this submitted article are our own and not an official position of the institution.
